# Barriers towards insulin therapy in type 2 diabetic patients: results of an observational longitudinal study

**DOI:** 10.1186/1477-7525-8-113

**Published:** 2010-10-04

**Authors:** Norbert Hermanns, Marina Mahr, Bernd Kulzer, Sören E Skovlund, Thomas Haak

**Affiliations:** 1 Research Institute of the Diabetes Academy Bad Mergentheim, Bad Mergentheim, Germany; 2 Novo Nordisk, Copenhagen, Denmark

## Abstract

**Background:**

The course of barriers towards insulin therapy was analysed in three different groups of type 2 diabetic patients. This observational longitudinal study surveyed a three-month follow-up.

**Methods:**

Participants in this study totalled 130 type 2 diabetic patients. The first subgroup was on insulin therapy at baseline (group 1: n = 57, age 55.6 ± 8.7 yrs, disease duration 12.7 ± 7.2 yrs, HbA1c 8.5 ± 1.6%) and remained on insulin at follow-up. Of an initial 73 insulin-naïve patients, 44 were switched to insulin therapy (group 2: age 58.1 ± 6.8 yrs, disease duration 7.7 ± 5.0 yrs, HbA1c 9.1 ± 1.7%) and 29 patients remained on an oral regimen (group 3: age 52.7 ± 10.7 yrs, disease duration 5.3 ± 4.6 yrs, HbA1c 8.3 ± 1.4%). Barriers towards insulin therapy were measured using the Insulin Treatment Appraisal Scale (ITAS). As generic instruments of health related quality of life patients completed also the Problem Areas of Diabetes Questionnaire (PAID), the WHO-5 Well-Being Scale (WHO-5), the Centre for Epidemiologic Studies Depression Scale (CES-D) and the Trait Version of the State Trait Anxiety Inventory (STAI) at baseline and at three-month follow-up.

**Results:**

At the three-month follow-up, HbA1c had improved in all three groups (7.7 ± 1.2% vs. 7.1 ± 1.1% vs. 6.7 ± 0.8%). The course of negative appraisal of insulin therapy was significantly different in the three groups (p > .003): the ITAS score increased in patients remained on oral antidiabetic drugs (51.2 ± 12.2 to 53.6 ± 12.3), whereas it decreased in patients switched to insulin therapy (49.2 ± 9.8 to 46.2 ± 9.9) or remained on insulin treatment (45.8 ± 8.3 to 44.5 ± 8.0). Diabetes-related distress, trait anxiety, and well-being, showed a similar course in all three groups. The depression score improved significantly in patients switched to insulin treatment compared with patients remaining on insulin therapy.

**Conclusions:**

In summary, this study suggests that a negative appraisal of insulin treatment is modifiable by the initiation of insulin therapy. This finding indicates that barriers to insulin are a rather temporary than a stable phenomenon.

## Background

Poor glycaemic control is a risk factor for the development of diabetes-specific complications in diabetic patients. Many type 2 diabetic patients require insulin therapy after several years of disease duration in order to maintain good glycaemic control and prevent complications. But many type 2 diabetic patients do not receive insulin therapy in a timely manner because of a negative appraisal of this treatment option [1].

Patients' negative attitudes towards starting insulin therapy are based on their beliefs that the need for insulin therapy indicates a greater severity of the disease and proves their failure to self-manage the diabetes adequately. Worries about painful injections and the risk of severe side effects such as hypoglycaemia are also very common among type 2 diabetic patients. Some type 2 diabetic patients fear that insulin therapy is too difficult to manage in everyday life and is associated with social stigma [2]. In the literature the above-described phenomenon is called psychological insulin resistance [3,4].

Cross-sectional data demonstrate that barriers to insulin therapy are higher in insulin-naïve type 2 diabetic patients than in insulin-treated type 2 diabetic patients [2,5]. However, from cross-sectional analysis it is difficult to decide whether barriers to insulin therapy exists mainly temporary and reduce by the experience of insulin therapy or whether it is a selection factor caused by the circumstance that only type 2 diabetic patients with a more positive appraisal of insulin therapy will accept this treatment option. From a clinical perspective, each explanation would require a different therapeutic approach. In the first case, diabetes education or better instructions about insulin treatment could be powerful tools to help type 2 diabetic patients coping better with the challenges of insulin therapy and changing their negative appraisal of this treatment option. In the latter case, new types of insulin or different forms of insulin applications (e.g. inhaled insulin) might be helpful to reduce barriers to insulin therapy and encourage a greater proportion of type 2 diabetic patients to use this powerful treatment option. Thus, clearly, longitudinal data on the course of barriers towards insulin therapy after the initiation of insulin therapy are needed to address these issues.

It can also be expected that negative appraisal of insulin therapy in combination with a need for initiation of insulin therapy has a negative impact on other quality of life aspects such as diabetes-related distress, symptoms of depression and anxiety, and psychological well-being among diabetic patients [2,5].

In this observational study we compared the barriers to insulin therapy and more generic quality of life aspects among insulin-treated and insulin-naïve type 2 diabetic patients who had poor glycaemic control at baseline and three months after an intensification of diabetes treatment. In a subgroup of insulin-naïve type 2 diabetic patients, insulin therapy was initiated.

## Methods

Participants in this observational longitudinal study were type 2 diabetic patients who were referred by general practitioners to practices of diabetologists or to the Diabetes Centre Mergentheim. The main reason for referral was unsatisfactory glycaemic control according to the guideline of the German Diabetes Association [6].

### Inclusion criteria of the study

• Poor glycaemic control (HbA1c > 6.5% or blood glucose excursions)

• Age 18-75 years

• Ability to understand the German language

• Informed consent given

### Exclusion criteria

• Severe life-threatening disease according to the judgement of a diabetologist

• Guardianship

The study was approved by an ethics committee. All patients declared informed consent to participate in this observational study.

### Measures

Appraisal of insulin therapy was measured by the Insulin Treatment Appraisal Scale (ITAS) [2]. The ITAS, which was designed to assess attitudes towards insulin treatment in type 2 diabetic patients, consists of 20 items. Subjects are requested to indicate on a 5-point Likert scale to what extent they agree with each statement, from "strongly disagree" to "strongly agree". The ITAS is a two-dimensional instrument, with "appraisal of insulin therapy" as a single underlying construct. The instrument permits the calculation of a total score and two subscale scores that measure positive (4 items) and negative (16 items) attitudes towards insulin treatment. Example items representing a positive attitudes towards insulin therapy are: "Taking insulin helps to prevent complications of diabetes" or "Taking insulin helps to maintain good control of blood glucose". Items like "Taking insulin means I have failed to manage my diabetes with diet and tablets" or "Managing insulin injections takes a lot of time and energy" are examples representing a negative attitude towards insulin therapy. The psychometric properties of the ITAS were confirmed recently. The reliability of the total scale was Cronbach's α = .89 (negative appraisal scale Cronbach's α = .90, positive appraisal scale Cronbach's α = .68) and can be regarded as highly satisfactory [2].

As generic measures of emotional well-being and emotional distress, established depression-, anxiety-, and well-being scales were used. Patients completed the German version of the Centre for Epidemiologic Studies Depression Scale (CES-D) [7,8] to assess depressive symptoms. The CES-D has a scale range between 0 and 60; higher scores indicate higher levels of depressive symptoms. Anxiety symptoms were measured by the trait version of the State-Trait-Anxiety-Inventory (STAI) [9,10]. This questionnaire has a scale range between 20 and 80. Higher scores on the Trait-STAI represent higher levels of trait anxiety symptoms. The WHO-5 well-being scale was used to measure well-being [11]. The WHO-5 contains five items, which are all positively worded. A maximum score of "25" indicates optimal well-being, whereas a score of "0" indicates minimal well-being. Diabetes-specific distress was assessed by the German version of the Problem Areas in Diabetes Scale (PAID) [12]. The PAID questionnaire consists of 20 items. Each item can be rated on a 5-point Likert scale ranging from "0" (no problem) to "4" (serious problem). According to the recommendation of the test's authors, the PAID scores were transformed to a 0-100 scale, with higher scores indicating more serious emotional problems. The original scale has proved its validity and reliability [13].

Glycaemic control was measured by HbA1c through use of high pressure liquid chromatography. The normal range of HbA1c is 4.1% - 6.1%.

### Statistical analysis

Continuous data were analysed by use of parametric methods, and categorical data were analysed by Chi-Square tests. For comparisons of three groups, analyses of variance were used. The analysis of differences between baseline and follow-up measurements was adjusted for baseline values through use of covariance analysis (ANCOVA). Post hoc tests were performed in case of an overall significant effect.

## Results

### Baseline characteristics

Participants in this study totalled 130 type 2 diabetic patients, of whom 73 were insulin-naïve and 57 were insulin-treated. As shown in table 1 insulin-treated and insulin-naïve patients were of a comparable relatively young age and had a similar gender distribution, with fewer women than men. Glycaemic control was rather poor in both groups, as could be expected from the inclusion criteria. Insulin-naïve patients demonstrated a significantly shorter disease duration and had significantly fewer diabetes-associated complications. As could be expected from previous findings, insulin-naïve patients had a significantly more negative appraisal of insulin therapy than patients who already were being treated with insulin. The insulin-treated patients demonstrated a tendency towards higher depression scores (p < .10). On the remaining scales, insulin-treated type 2 diabetic patients had higher scores, a result that indicates a lower health related quality of life. However, these differences failed to reach significance level.

**Table 1:** Baseline characteristics

	Insulin-naïve type 2 diabetic patients (n = 73)	Insulin-treated type 2 diabetic patients (n = 57)	p
**Mean age ± SD (yrs)**	56.0 ± 8.9	55.6 ± 8.7	.824

**% female (number)**	31.5 (23)	38.6 (22)	.399

**Mean disease duration ± SD (yrs)**	6.8 ± 5.0	12.7 ± 7.2	< .001

**Mean HbA1c ± SD (%)**	8.8 ± 1.6	8.5 ± 1.6	.455

**Mean BMI ± SD (kg/m^2^)**	32.6 ± 6.0	35.7 ± 7.9	.0.17

**# of complications ^1^**	0.6 ± 1.0	1.4 ± 1.3	< .001

**Mean ITAS ± SD (total score)**	50.0 ± 10.8	45.8 ± 8.3	.013
**Mean negative ITAS ± SD**	41.3 ± 10.4	37.4 ± 7.6	.016
**Mean positive ITAS ± SD**	9.9 ± 2.3	9.4 ± 1.9	.175

**Mean PAID ± SD**	31.4 ± 18.3	28.2 ± 14.1	.257

**Mean WHO-5 ± SD**	14.7 ± 6.3	12.9 ± 6.4	.115

**Mean CES-D ± SD**	14.4 ± 9.7	17.8 ± 10.9	.072

**Mean Trait Anxiety ± SD**	39.9 ± 11.3	41.6 ± 8.4	.334

^1^Retinopathy, nephropathy, diabetic neuropathy, diabetic foot syndrome, coronary heart disease, stroke, arterial occlusive disease

Of the patients receiving insulin therapy, 27 (47.4%) had an insulin monotherapy and 30 patients received a combination therapy of insulin and oral antidiabetic medication. The two subgroups did not differ with regard to baseline HbA1c (8.6 ± 1.9% vs. 8.5 ± 1.5%, p = .74).

### Follow up results

Table 2 shows the baseline characteristics of patients switched to insulin vs. patients who remained on treatment with oral antidiabetic agents. Patients who were switched to insulin therapy were significantly older, had a significantly longer diabetes duration, and had more diabetes complications than patients who remained on an oral diabetes regimen. Patients who were switched to insulin therapy had a higher HbA1c level than patients who remained on treatment with antidiabetic drugs. As reported in table 3 there were no significant differences on the ITAS total score between patients who remained on an oral regimen and patients who were switched to insulin therapy (pairwise comparisons between these two groups p = .674).

**Table 2:** Baseline characteristics of patients switched to insulin vs. patients remained insulin naïve.

	Remained insulin-naïve n = 29	Switched to insulin treatment n = 44	p
**Mean age ± SD (yrs)**	52.7 ± 10.7	58.1 ± 6.8	.010

**% female (number)**	31.0 (9)	31.8 (14)	.945

**Mean disease duration ± SD (yrs)**	5.3 ± 4.6	7.7 ± 5.0	.040

**Mean HbA1c ± SD (%)**	8.3 ± 1.4	9.1 ± 1.7	.027

**# of complications ^1^**	0.2 ± 0.5	0.9 ± 1.2	.002

^1 ^Retinopathy, nephropathy, diabetic neuropathy, diabetic foot syndrome, coronary heart disease, stroke, arterial occlusive disease

**Table 3:** Baseline and follow-up results in the three different treatment groups

	Patients who remained insulin-naïve	Patients switched to insulin treatment	Patients who remained on insulin therapy	p between groups
HbA1c (%)				

Baseline	8.3 (± 1.4)	9.1 (± 1.7)	8.6 (± 1.7)	.078
Endpoint	6.7 (± 0.8)	7.1 (± 1.1)	7.7 (± 1.2)	< .001^c^
Change	-1.6 (± 1.7)*	-1.8 (± 1.8)*	-0.9 (± 1.7)*	< .001^1bc^

BMI (kg/m^2^)				

Baseline	32.9 (± 6.5)	32.4 (± 5.8)	35.7 (± 7.9)	.051
Endpoint	31.2 (± 6.6)	31.3 (± 5.2)	35.0 (± 7.9)	.010^bc^
Change	-1.7 (± 1.5)*	-1.1 (± 1.9)*	-0.7 (± 2.6)*	.054^1^

ITAS (total score)				

Baseline	51.2 (± 12.2)	49.2 (± 9.8)	45.8 (± 8.3)	.040 ^c^
Endpoint	53.6 (± 12.3)	46.2 (± 9.9)	44.5 (± 8.0)	< .001^bc^
Change	2.4 (± 14.8)^ns^	-3.0 (± 7.0)*	-1.3 (± 8.0) ^ns^	.003^1ac^

Negative ITAS				

Baseline	41.8 (± 12.2)	41.0 (± 9.1)	37.4 (± 10.4)	.063
Endpoint	43.4 (± 11.8)	37.9 (± 8.7)	35.7 (± 7.4)	.002^bc^
Change	1.6 (± 15.0) ^ns^	-3.1 (± 6.5)*	-1.7 (± 7.3) ^ns^	.006^1ac^

Positive ITAS				

Baseline	10.4 (± 2.5)	9.5 (± 2.2)	9.4 (± 1.9)	.098
Endpoint	11.0 (± 2.8)	9.4 (± 2.2)	9.6 (± 2.0)	.008^bc^
Change	0.6 (± 3.0) ^ns^	-0.1 (± 2.2) ^ns^	0.2 (± 2.4) ^ns^	.042^1ac^

PAID				

Baseline	27.7 (± 17.8)	33.8 (± 18.4)	28.2 (± 14.1)	.166
Endpoint	21.8 (± 13.9)	26.5 (± 15.6)	24.6 (± 16.9)	.384
Change	-5.9 (± 15.8) ^ns^	-7.3 (± 14.9) *	-3.6 (± 15.1) ^ns^	.285^1^

WHO-5				

Baseline	16.4 (± 6.0)	13.6 (± 6.3)	12.9 (± 6.4)	.056
Endpoint	16.7 (± 5.6)	15.6 (± 5.8)	13.9 (± 6.0)	.129
Change	0.3 (± 5.7) ^ns^	2.0 (± 4.9) *	1.0 (± 5.7) ^ns^	.389^1^

CES-D				

Baseline	11.7 (± 8.2)	16.1 (± 10.2)	17.8 (± 10.9)	.039^c^
Endpoint	10.8 (± 7.9)	11.9 (± 9.8)	16.4 (± 9.8)	.019
Change	-0.9 (± 7.1) ^ns^	-4.2 (± 6.0) *	-1.4 (± 8.5) ^ns^	.045^1b^

Trait Anxiety				

Baseline	37.7 (± 9.7)	41.3 (± 12.0)	41.6 (± 8.4)	.227
Endpoint	36.4 (± 8.3)	40.2 (± 10.6)	40.7 (± 9.9)	.138
Change	-1.3 (± 5.9) ns	-1.1 (± 8.2) ns	-0.9 (± 7.2) ns	.622^1^

Values are means (± SD); ^1 ^adjusted for baseline; ^a ^= p < .05 between "patients who remained insulin-naïve" and "patients switched to insulin treatment"; ^b ^= p < .05 between "patients switched to insulin treatment" and "patients who remained on insulin therapy"; ^c ^= p < .05 between "patients who remained insulin-naïve" and "patients who remained on insulin therapy"; * significant within comparison (p < .05), ^ns ^non-significant within comparison (p > .05)

All patients who were on insulin therapy at baseline remained on this treatment. The HbA1c level in the patients remaining on insulin treatment fell significantly. Interestingly, this outcome was achieved by increasing the proportion of patients who received a combination treatment of oral antidiabetic agents and insulin. The proportion of patients on combination therapy with oral antidiabetic agents and insulin rose from 47.4% to 71.9% (McNemar test p < .05). The mean daily insulin dose decreased from 0.88 ± 0.62 IU/kg to 0.63 ± 0.44 IU/kg (p < .01). Body weight was also slightly, but significantly reduced.

Of the patients on oral antidiabetic medication at baseline, 44 (60.3%) were switched to insulin therapy. The decision was made by the treating diabetologist, based on clinical judgement. No patient in this study rejected the insulin therapy option.

The patients switched to insulin therapy received, on average, 0.28 ± 0.23 IU/kg and injected insulin 1.9 ± 1.4 times a day on average. HbA1c improved significantly in these patients. Interestingly, BMI was also significantly reduced (see table 3).

In the patients who remained on oral antidiabetic medication, HbA1c also improved significantly.

Although in all three groups there was a significant within effect on HbA1c in the follow-up period, the improvement of glycaemic control was significantly greater in patients who were switched to insulin therapy or remained on an oral regimen than in patients who remained on insulin therapy.

Barriers towards insulin therapy developed significantly different among the three groups. In patients who remained on an oral regimen, the negative appraisal of insulin therapy increased, whereas in patients who were switched to insulin therapy the negative appraisal of insulin therapy was reduced to the level of patients who remained on insulin therapy.

The same pattern of change was present regarding the subscale of negative appraisal of insulin treatment. In the subscale of positive appraisal of insulin therapy the scores were rather stable in all three patient groups.

In patients on an oral regimen at baseline, there was a remarkable improvement of diabetes-related distress regardless of whether those patients remained on an oral regimen or were switched to insulin treatment. Patients who remained on insulin treatment improved slightly. However, there was no significant overall change in diabetes-related distress in the three treatment groups.

General well-being improved slightly in all three groups, but there was no significant difference among these patients groups.

Depressive symptoms were significantly reduced in patients who were switched to insulin therapy compared with patients who remained on insulin therapy.

There was no significant effect on trait anxiety during the follow-up period.

## Discussion

At baseline, more barriers to insulin therapy were demonstrated by insulin-naïve type 2 diabetic patients compared with insulin-treated type 2 diabetic patients. This result is in line with previous findings of cross-sectional studies [2-5]. However, cross-sectional data are difficult to interpret. It is difficult to decide whether the lower level of negative appraisal of insulin therapy among insulin-treated type 2 diabetic patients is a consequence of adaptation to the demands of insulin treatment or whether a selection bias is mainly responsible for this finding. Patients who have a less negative appraisal of insulin treatment might be more likely to accept insulin treatment than patients who have a more objections against this treatment option.

This study provides longitudinal data about the course of negative appraisal of insulin in insulin-naïve and insulin-treated type 2 diabetic patients. Of the insulin-naïve patients, 60% were switched to insulin treatment. At baseline, those type 2 diabetic patients who were switched to insulin therapy and those patients who remained on an oral regimen did not differ with regard to their appraisal of insulin treatment. Thus a selection bias, meaning that only patients who had lower barriers to insulin therapy were switched to insulin treatment, seems unlikely.

At the three-month follow up, it could be demonstrated clearly that barriers to insulin therapy increased in patients who remained on an oral regimen, whereas negative appraisal of insulin treatment was reduced in patients who were switched to insulin therapy. The negative appraisal of insulin treatment in patients who were switched to insulin therapy was reduced to the level of patients already treated with insulin.

Therefore, it seems reasonable to assume that patients who are exposed to insulin therapy acquire new skills regarding how to handle insulin and change their appraisal of this treatment option. These patients may accommodate to this treatment alternative and reduce their barriers to insulin therapy.

One strength of this study is that in addition to negative appraisal of insulin treatment, a broad assessment of more generic psychological variables such as well-being, diabetes-related distress, anxiety, and depressive symptoms were longitudinally assessed. Except for a significant effect on depression, there was no specific impact of the subsequent diabetes treatment on anxiety symptoms, diabetes-related distress, or psychological well-being. These findings might indicate that negative attitudes regarding insulin treatment is a rather specific barrier to this treatment option and is not strongly associated with general aspects of health related quality of life. This idea is corroborated by the finding that patients who remained on an oral regimen had the lowest depression scores at baseline as well as at follow-up, although their negative appraisal of insulin treatment increased.

Patients who remained on insulin treatment and patients who were switched to insulin treatment had more late complications than insulin-naïve patients. It is well known that complications of diabetes are associated with depression [14,15]. Most patients who were switched to insulin treatment experienced remarkably improved glycaemic control. This outcome might have had a specific antidepressive effect, which could explain the significantly greater reduction of depressive symptoms in this group.

There are also some limitations of this study. The study is observational, meaning that the groups were not randomised. The decision who remained on an oral regimen and who was switched to insulin was at the discretion of the clinicians. Although this clinical judgement proved to be effective with regard to the glycaemic control achieved during follow-up, a selection bias cannot be excluded.

The sample size is rather small; thus a lack of power could be responsible for the fact that the effect of the diabetes treatment at follow-up could not be assessed with respect to more generic variables such as anxiety, diabetes-related distress, and well-being.

The follow-up period is rather short; longer follow-up period is needed to evaluate if reducing barriers to insulin therapy is maintained, further reduced or increased over time.

## Conclusions

Nevertheless, in summary, this study demonstrates that negative appraisal of insulin treatment is modifiable by the initiation of insulin therapy. This finding indicates that barriers to insulin treatment is a benign, temporary phenomenon instead of an unvarying patient characteristic. Future studies should address if identifying and addressing patients concerns about insulin therapy can help to improve long-term adaptation to insulin therapy.

## Competing interests

This study was supported by an unrestricted grant of Novo Nordisk. NH and BK are members of the German DAWN advisory board supported by Novo Nordisk.

## Authors' contributions

NH participated in the design of the study, performed the statistical analysis and drafted the manuscript. MM participated in the design of the study and collected the data. BK participated in the design of the study and helped to draft the manuscript. SES has been involved in revising the manuscript critically for important intellectual content. TH participated in the design of the study and coordination and helped to draft the manuscript. All authors read and approved the final manuscript.
